# Researchers experience of using the regulatory affairs information system (RAIS) in strengthening research compliance in a large research program: A case study of the Infectious Diseases Institute (IDI) in Uganda

**DOI:** 10.1093/jamiaopen/ooac059

**Published:** 2022-07-14

**Authors:** Sylvia Nabukenya, Stephen Okoboi, Vivian Nakate, Adelline Twimukye, Bruce Opio, Barbara Castelnuovo

**Affiliations:** Research Department, Infectious Diseases Institute, College of Health Sciences, Makerere University, Kampala, Uganda; Research Department, Infectious Diseases Institute, College of Health Sciences, Makerere University, Kampala, Uganda; Research Department, Infectious Diseases Institute, College of Health Sciences, Makerere University, Kampala, Uganda; Research Department, Infectious Diseases Institute, College of Health Sciences, Makerere University, Kampala, Uganda; Research Department, Infectious Diseases Institute, College of Health Sciences, Makerere University, Kampala, Uganda; Research Department, Infectious Diseases Institute, College of Health Sciences, Makerere University, Kampala, Uganda

**Keywords:** regulatory affairs, information system, research compliance

## Abstract

**Objective:**

The aim of this study was to explore researchers’ experience of using the regulatory affairs information system (RAIS) in strengthening research compliance to national ethics guidelines through tracking ethics and regulatory approvals for research projects at the Infectious Diseases Institute.

**Methods:**

We conducted a cross-sectional study using purposive sampling of 50 participants who were principal investigators (PI) and study coordinators (SC) of active projects between November 2019 and January 2020. Only 36 of them responded to the survey. We also conducted 12 key informant interviews among PI, SC, and research management at the Institute. We used STATA 13 to analyze responses to the survey. The interviews lasted between 20 and 30 min. We used NVivo 10 software to manage the transcripts and generation of themes.

**Results:**

Majority 19 (52.8%) of those who participated in the survey were study coordinators, 19 (52.8%) had participated in more than 5 research studies, 28 (90.3%) had ever received a notification from the RAIS and 26 (92.9%) submitted requests for renewal of their studies approvals to Ethics committees and regulatory bodies 4 weeks prior to expiration dates. The study also examined participants’ general understanding of the regulatory requirements and all were aware that RECs and NDA grant approval for a period of 1 year, and 35 (97.2%) that UNCST grants approval for the duration of the study. Three prominent themes; researchers’ experiences, benefits, and shortcomings of RAIS were generated from the key informant interviews.

**Discussion:**

Having experience in research coupled with a novel automated system provides a platform for a better understanding of research regulatory requirements, hence compliance to the national guidelines.

**Conclusion:**

Our case study demonstrates that supporting researchers and research institutions in low resource settings with an automated system in tracking expiration dates for research approvals can facilitate compliance to national ethics guidelines.

## INTRODUCTION

All research involving human participants is required to have prospective ethical approval from an independent Research Ethics Committee (REC). In Uganda this is enforced by the Uganda national guidelines for research involving humans participants, and other international guidelines such as the Nuremburg Code,^1^ Declaration of Helsinki,^2^ the Belmont Report,^3^ the Council for the International Organizations of Medical Sciences (CIOMS),^4^ and the International Convention for Harmonization’s initiative on Good Clinical Practice (GCP). RECs have a primary role of protecting research participants and their communities against any form of harm and exploitation that may result from research activities and procedures.

The Uganda national guidelines for human research define research as any type of systematic investigation, testing, and evaluation, designed to contribute to generalizable knowledge. In Uganda, researchers must seek for a prospective ethical approval from any accredited Research Ethics Committee (REC), which is granted for a period of 1 year, and regulatory approval from the Uganda National Council for Science and technology (UNCST) granted for the duration of the study (but not more than 5 years) with submission of annual progressive reports. Researchers proposing to conduct clinical trials involving investigational medicinal products must seek approval from the National Drug Authority (NDA) before commencement of study activities. NDA grants approval for a period of 1 year. Once ethics or regulatory approvals expire, researchers submit an annual report of study activities and an application for renewal of approval to RECs or regulatory bodies.

Over the years, the number of research studies at the Infectious Diseases Institute (IDI) has greatly increased with an average of 65 studies annually. The IDI is a Ugandan not-for-profit organization established in 2002 under the College of Health Sciences, Makerere University. The IDI is one of the leading HIV research organizations in Uganda, and conducts clinical trials, observational studies, diagnostic studies, implementation science research, and support students’ research. The IDI has so far housed over 260 research studies.

**Figure 1.**
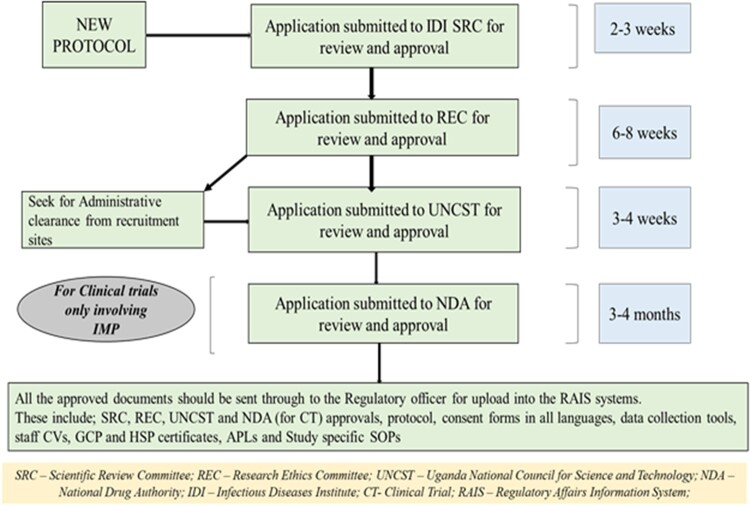
Regulatory affairs framework of the Infectious Diseases Institute (IDI).

All researchers intending to implement their research projects at the IDI must seek for institutional clearance from the IDI Scientific Review Committee before embarking in submission to the REC (see also Figure 1).

Due to the numerous activities, researchers are faced with challenges in tracking expiration dates of regulatory and ethics approvals. A study conducted by the UNCST showed that 25% of the studies monitored between 2007 and 2010 in Uganda continued their research activities with expired REC and or UNCST approvals.^5^

In 2015, we developed a centralized electronic system to track the entire regulatory process of research projects at the IDI. In this study, we aimed at exploring researchers’ understanding and experience of using a regulatory affairs information system (RAIS) developed at IDI. Uganda is a low resource setting and the use of such systems is not widely used in many research institutions.

## METHODS

### Study setting

The RAIS is a web based system, developed in house using a Net framework and runs on any operating system using a web browser such as “Google Chrome” and “Mozilla Firefox.” RAIS has several functionalities such as acting as a repository for research projects’ essential documents, fast access point for projects’ information and sending automated reminders to researchers and research management, which is the main focus of this article.

The Principal investigator obtains approvals from an accredited research ethics committee and regulatory bodies in writing after meeting all the necessary requirements. The Research Regulatory officer uploads into the RAIS the approval letters from the regulatory bodies and ethics committees, and the other approved documents like the protocol, consent forms in all languages and data collection tools Other documents are also uploaded: study staff contact information and training certificates (Good Clinical Practice [GCP], Human Subject Protection [HSP], Good Clinical Laboratory Practice [GCLP] certificates) and Annual practicing licenses (APL). Figure 1 illustrates the regulatory affairs framework of the IDI.

**Figure 2.**
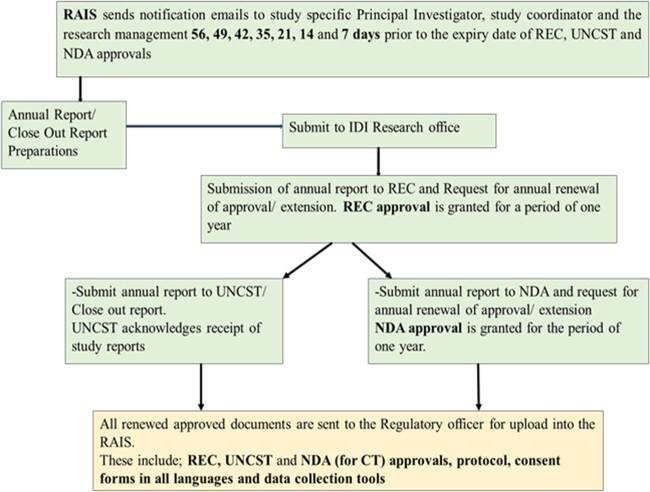
The role of the regulatory affairs information system (RAIS) in compliance to regulatory guidelines.

RAIS sends an automated “no-reply” email to the investigators, study coordinators or project managers and research management notifying need for renewal of their study’s approvals 56, 49, 42, 35, 28, 14, and 7 days before the expiration date. The investigator or designated person prepares the application package for renewal of approvals. The package is then sent to the Research Regulatory Officer for review and submission to the regulatory authorities (see Figure 2). Figure 2 illustrates the role of the RAIS in compliance to regulatory guidelines.

Upon receipt of renewed approval from the regulatory bodies, the Research Regulatory officer uploads all the essential documents stamped by the respective RECs into RAIS. This cycle is continuous throughout the lifecycle of the project.

### Participants and data collection

We adopted both quantitative and qualitative methods of data collection.

Principal investigators and study coordinators of active research projects were purposively sampled to participate in a 1-time survey (cross-sectional). They were contacted by email, which contained an information sheet about the study and a link to the survey comprising 3 sections. The first section contained questions on socio demographic characteristics, the second section comprised questions on participants’ understanding of regulatory requirements. The third section comprised questions on participants’ understanding of the RAIS functionality.

We also conducted face-to-face key informant interviews among principal investigators, study coordinators, and members of the IDI research management. The interviews aimed at gaining an in-depth understanding of their experience in using the RAIS to strengthen research compliance at the Institute. The interviews were guided by a set of open-ended questions to explore participants’ experiences, benefits, possible shortcomings, and strategies that can be used to improve the functionality of the RAIS. Questions asked include; what was it like to keep track of the ethics and regulatory approvals of research studies at the IDI? How often have you interfaced with the RAIS in regards to submission of progress reports on time? How has RAIS been of importance to you as an end user at the IDI? What the possible shortcomings of RAIS to you as an end user?

On average, interviews lasted between 20 and 30 min. Discussions between the researcher and the research participants were conducted in English and audio recorded.

Both qualitative and quantitative data were collected between November 2019 and January 2020. Participants selected were Principal investigators, study coordinators, and some members of the IDI Research management with vast experience and understanding of the RAIS functionality.

### Data analysis

Responses to the survey were analyzed using STATA 13. Frequencies and percentages were used to describe participants’ demographic characteristics and their responses. All audio-recorded interviews were transcribed verbatim. Two of the authors (SN and AT) developed a codebook based on emergent themes; disagreements were solved by consensus. Verified transcripts were imported into NVivo 10 software to manage and organize the data. Data analysis was conducted continuously throughout the study using a thematic approach. The first step of the analysis involved reading of all transcripts word by word to familiarize with the data. We then performed open line-by-line coding to generate the first set of codes. The codebook was then refined to identify themes in relation to participants’ experience of using the RAIS in strengthening research compliance. Themes were supported by representative quotes.

### Ethical considerations

This research was reviewed and approved by the Makerere University School of Biomedical Sciences Higher Degrees and Research Ethics Committee (SBS-HDREC No. 679) and the Uganda National Council for Science and Technology (SIR 18ES). Electronic consent was obtained from individuals prior to their participation in the survey and interviews. An information sheet containing the study procedures, benefits, and risks was provided to participants by email. Participants who voluntarily agreed to take part in the survey expressed their interest by clicking only “I agree to participate” before proceeding to the online survey. All participants were assured of confidentiality and access to all data collected from the survey and interviews is limited to research investigators.

## RESULTS

A total of 50 researchers had active projects; all were invited to participate and 36 (72%) responded to the online survey. Table 1 summarizes participants’ characteristics. The participants’ average age was 37 years, 20 (55.6%) were female and 19 (52.8%) were study coordinators. Of interest, 19 (52.8%) had joined IDI after 2015 and 24 (66.7%) had worked with IDI for less than 10 years. Majority 19 (52.8%) had participated in more than 5 research studies and 14 (38.9%) were involved in on observational studies.

**Table 1.** Characteristics of research participants

Variable	Frequency *N* (%), *N* = 36
Age	
<35 years	14 (38.9)
>35 years	22 (61.1)
Sex	
Female	20 (55.6)
Male	16 (44.4)
Number of years worked in research field	
<10 years	24 (66.7)
10 years and above	12 (33.3)
Role	
Principal investigator	17 (47.2)
Study coordinator	19 (52.8)
Year when staff joined IDI	
Before 2015	17 (47.2)
After 2015	19 (52.8)
Highest level of education qualification achievement	
Bachelor’s degree	7 (19.4)
Master’s degree	23 (63.9)
PhD	5 (13.9)
Other	1 (2.8)
Area of research	
Administration	1 (2.8)
Clinical trials	12 (33.3)
Diagnostic	4 (11.1)
Observational studies	14 (38.9)
Other	5 (13.9)
Number of research studies	
5 or less	17 (47.2)
Above 5 studies	19 (52.8)

*N*: number.

### Participants’ understanding of regulatory requirements

**Figure 3.**
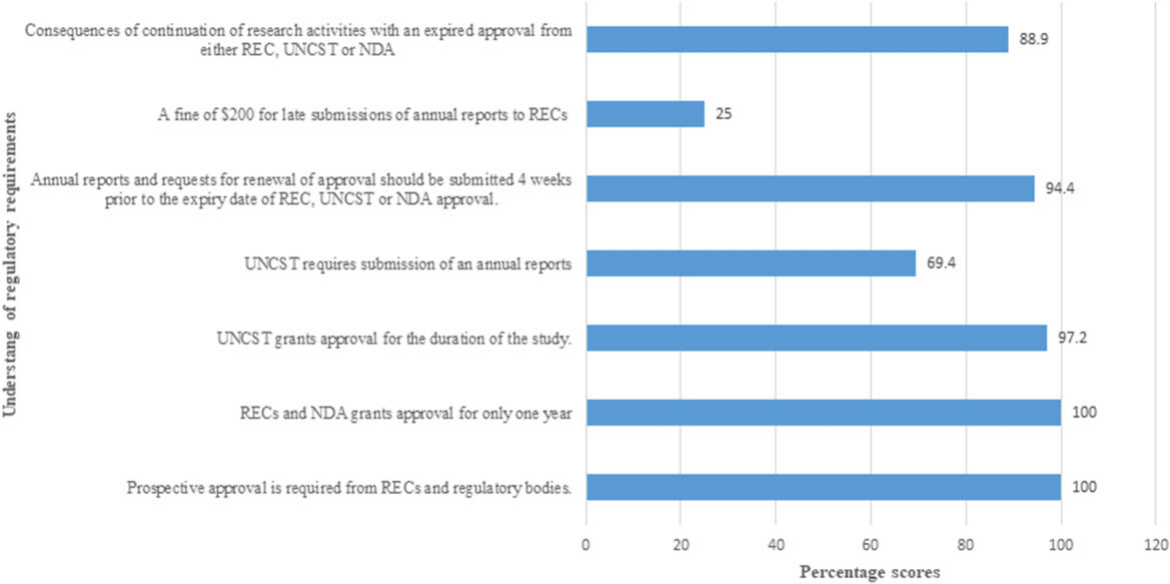
Participants’ understanding of regulatory requirements.

Figure 3 shows participants’ understanding of regulatory requirements. All the participants were aware of the requirement for prospective approvals from Research ethics committee and regulatory bodies. All were aware that RECs and NDA grant approval for a period of 1 year, and 35 (97.2%) that UNCST grants approval for the duration of the study. Only 25 (69.4%) were aware of submission of annual reports to UNCST and 34 (94.4%) were aware that annual reports and application for renewal of approvals should be submitted 4 weeks before the expiration. Only 9 (25.0%) were aware of a fine of $200 USD for late submission of annual reports to RECs. Thirty-two (88.9%) knew about the consequences of continuation of research activities with an expired approval from the regulatory bodies.

### Participants’ understanding of the RAIS functionality

Among the 36 participants who responded to the survey, 31 (86.1%) had heard about RAIS and were aware of its functionality. However, all the 5 participants who had never heard about RAIS expressed interest in learning about RAIS and its functionality. Table 2 describes participants understanding of the RAIS functions.

**Table 2.** Participants understanding of the RAIS functionality

	Frequency *N* (%)
Ever received a notification email from the RAIS reminding you about study’s expiration approval dates	
Yes	28 (90.3)
No	3 (9.7)
Estimated number of times ever received a RAIS notification	
3 times and more	18 (64.3)
Less than 3 times	10 (35.7)
Submitted annual reports and requests for renewal of approval 4 weeks prior to expiration dates of RECs, UNCST, or NDA	
Yes	26 (92.9)
No	2 (7.1)
RAIS has supported to comply with the regulatory requirements	
Agree	26 (92.9)
Neither agree or disagree	1 (3.55)
Disagree	1 (3.55)

*N*: number.

### Themes generated from interviews

Key informant interviews were conducted among 12 participants. Majority 11(92%) were >35 years, 7 (58%) were female and 2 (were non-Ugandan). Participants had varying roles; 5 (42%) were study coordinators, 3 (25%) were Principal investigators, and 4 (33%) were representatives of the IDI research management. A half of them 6 (50%) had worked in IDI for over 5 years, and had participated in more than 5 studies.

Table 3 shows 3 prominent themes that were generated from the key informant interviews.

**Table 3.** Themes generated from interviews

Theme	Description
Experience	Researchers’ experience of tracking the expiration dates of approvals from the Research ethics committees and regulatory bodies’ before and after the implementation of the RAIS.
Benefits	Ways in which RAIS has supported research teams in compliance to national guidelines and institutional policies
Shortcomings	Current and foreseeable limitations of the RAIS

### Researchers experience of tracking expiration dates of approvals from the research ethics committees and regulatory bodies

Before the implementation of RAIS, researchers at IDI used various ways of keeping track of the expiration dates of their studies’ approvals. These include excel sheets, outlook calendars, and reminders from funders.

*As a study coordinator overseeing three projects, I relied on an excel sheet. The complexity in tracking these approval dates was, it is not only one approval but rather I had to track the expiration dates for REC, UNCST,and NDA approvals. More so, all the approvals had different dates. And also, despite my other responsibilities, I still had to remember to check on my manually generated excel sheet. [Study coordinator #7]*

Researchers had to adjust their ways of tracking their approvals’ expiration dates through adopting the electronic reminders. The notification reminders are often followed by several emails from the Regulatory team.

*I have received quite a number of notifications from RAIS for my two studies that am currently a PI [Principal investigator]. Also, research office helps add a follow up reminder email for action. I must admit that it took me a while to adjust to an electronic system. But I must say, the RAIS notifications have kept me in check especially when I have travelled outside of Uganda. [Principal Investigator #4]*

### Benefits of RAIS to the principal investigators, study coordinators, and research administration

The timely submission of applications for renewal of approvals helps prevent disruptions of study activities, avoid penalties for late submissions, and support the ethical conduct of research activities by ensuring approvals are current.

*My study has a duration of five years and I received UNCST approval for five years. We have been submitting just annual reports to UNCST, it skipped our [research team of this study] minds that our UNCST approval was about to expire until we received the RAIS notifications about the UNCST expiration. [Study coordinator #10]*

Furthermore, the RAIS acts as a hub for all research projects’ documentation. On several occasions, research funding agencies, sponsors, and collaborators often request for key performance indicators of research projects that have been conducted at the IDI in the past. The retrieval of information has been made faster by the RAIS.

*RAIS is a fast access point when I want to find information on studies with similar designs and collaborators. This information is usually very helpful in grants writing and protocol development. [Principal investigator #5].*

The volume of research projects registered at the IDI has gradually increased overtime. RAIS assists in identification of available and missing essential documents for all studies. RAIS provides timely updates on the number of active and closed studies within a particular timeframe to facilitate adequate planning of available resources in the IDI Research department.

*The RAIS has been used as a management tool in streamlining workflows of regulatory compliance in the Research department of IDI and cost effective in the long run. With the high volume of research projects registered at IDI, it would be impossible to effectively serve our researchers’ regulatory needs without a system like RAIS [Representative from IDI Research management #11]*

### Current and foreseeable shortcomings of the RAIS

The fear of a system breakdown may be a big challenge especially if there is no backup plan. Interviewees also highlighted the challenge of the automated email notifications to be received in junk or spam folders, which are not often checked by researchers.

*I still have fear of over reliance on automated systems because it brings about false confidence during the times it is up and running but once it shuts down for some period of time, it is a big challenge. [Principal investigator #1]*

Interviewees also expressed concerns about the possibility of system breakdown due to overload or hacking.

*I am always skeptical about systems software because we sometimes not have much control over who has the rights over our data and what measures are in place to secure our data. Even when the server is in-house, we still don’t have guarantee that it can’t be hacked into…. [Study coordinator #8]*

## DISCUSSION

Ensuring researchers’ compliance to ethical standards of conducting human based research is a responsibility of research institutions as described in the UNCST guidelines.^6^ Research institutions ensure compliance through conducting workshops and training staff and students on best practices of responsible conduct of research. In addition, they set up research ethics committees with a goal of protecting the rights, safety, and welfare of research participants. This is achieved through reviewing research protocols and conducting regular and impromptu monitoring visits for noncompliance.^7^^,^^8^ Other than these strategies, we are not aware of any automated system being used in Ugandan institutions to strengthen compliance to ethics guidelines. We found that the researchers at IDI had an understanding of the regulatory requirements with 90% being aware of most of the components described in the survey tool. This may be due to several reasons. First, a substantial portion (52.8%) were well-experienced and had participated in more than 5 research studies. Second, the institution provides training and that is likely one of the reasons that compliance is relatively good at the IDI. Only 69.4% were aware of submission of annual reports to UNCST that may be because UNCST provides approval for the duration of the research project and so a number of investigators may forget to submit their annual reports since they still have valid approval from UNCST.

Although few participants (25%) were aware of the 200 USD fine payable for late submission of annual reports and application for renewal of approval, this could be positively interpreted because it may imply that the majority were never fined, or otherwise they would have been aware of it.

The implementation of the RAIS has streamlined the regulatory process at the IDI. Ninety percent of the researchers had ever received an email reminder about the expiration dates of their approvals and 58% had received the reminder more than 3 times before submission of annual reports and applications for renewal of study approvals. This may have contributed to 90% submission of annual reports and requests for renewal of approval 4 weeks prior to expiration dates of RECs, UNCST, or NDA, as 93% of the researchers admitted that RAIS supported timely regulatory activities. By further investigating, we discovered that the studies of the 3 researchers who had never received RAIS notifications had a duration less than 1 year and therefore, the approval was still in the valid period of approval. This is reassuring in view of the reliability of the RAIS system.

User resistance to information systems implementation has been identified as a salient reason for the failure of new systems.^9^ Complete trust of electronic systems to have the ability to perform tasks appropriately and efficiently so as to satisfy the end users is sometimes unachievable.^10^ Some researchers also expressed fears of reliance on an electronic system for updates especially in case of a system breakdown. Fears or uncertainties about the future of information systems have been reported in low resource settings.^11^ These have been attributed to several reasons such as inadequate infrastructure, scarcity of human resources, lack of information and communication technological (ICT) skills, resistance to change and lack of policies governing access, and data sharing.^11^ To overcome this challenge, there will be continuous training for assurance of safety of information to staff.

Findings from this study may not be generalizable since they represent a single institution, which is a center of Excellence in HIV care and research in Uganda. However, our study demonstrates that even in resource settings, it is feasible to build an in house sustainable and effective automated system that is generally acceptable by the users, and offer appropriate training on national regulatory requirements. Another limitation is that only 70% of the researchers responded to the invite to participate in this survey; however, this proportion is generally acceptable for surveys of this nature.^12^ Additionally, according to the characteristics of the respondents, we feel that all categories of researchers at our Institute were well-represented.

## CONCLUSION

Our study demonstrates that regular training provided at the institute coupled with an automated regulatory system has supported researchers to comply with the guidelines for research involving human participants at our institution.

Since RAIS has a modular design that enables plug-ins to be developed, we will explore feasibility of expanding its functionality for more regulatory functions, particularly hosting electronic regulatory binders that will allow for remote study monitoring.

## FUNDING

This research received no specific grant from any funding agency in the public, commercial, or not-for-profit sectors.

## AUTHOR CONTRIBUTIONS

SN, the corresponding author, contributed to the conception and design of the work, acquisition and interpretation of data, drafting and revising the work critically for important intellectual content and provided final approval of the version to be published. SO contributed to the design of the work, revising the work critically for important intellectual content and provide final approval of the version to be published. VN and AT contributed to the analysis, interpretation of data for the work, drafting and revising the work critically for important intellectual content and provided final approval of the version to be published. BO contributed to the conception and design of the work, revising the work critically for important intellectual content and provided final approval of the version to be published. BC contributed to the design of the work, revising the work it critically for important intellectual content and provide final approval of the version to be published. All the authors agree to be accountable for all aspects of the work in ensuring that questions related to the accuracy or integrity of any part of the work are appropriately investigated and resolved.

## References

[1] Moreno JD , Schmidt U, Joffe S. The Nuremberg code 70 years later. JAMA 2017; 318 (9): 795. 28817743 10.1001/jama.2017.10265

[2] Ndebele P. The Declaration of Helsinki, 50 years later. JAMA 2013; 310 (20): 2145–6. 24141794 10.1001/jama.2013.281316

[3] Sims JM. A brief review of the Belmont report. Dimens Crit Care Nurs 2010; 29 (4): 173–4. 20543620 10.1097/DCC.0b013e3181de9ec5

[4] CIOMS. International Ethical Guidelines for Health-Related Research Involving Humans. 2016. 10.1001/jama.2016.18977 27923072

[5] Ochieng J , Ecuru J, Nakwagala F, Kutyabami P. Research site monitoring for compliance with ethics regulatory standards: review of experience from Uganda. BMC Med Ethics 2013; 14 (1): 23. 23738971 10.1186/1472-6939-14-23 PMC3683324

[6] UNCST. Uganda National Council for Science and Technology: National Guidelines for Research Involving Humans as Research Participants. Kampala, Uganda. 2014. https://uncst.go.ug/

[7] Hyder AA , Ali J, Hallez K, White T, Sewankambo NK, Kass N. Exploring institutional research ethics systems: a case study from Uganda. AJOB Empir Bioeth 2015; 6 (3): 1–14. 10.1080/23294515.2014.981316 PMC4652948 26594648

[8] Ochieng J , Mwaka E, Kwagala B, Sewankambo N. Evolution of research ethics in a low resource setting: a case for Uganda. Dev World Bioeth 2020; 20 (1): 50–60. 29958330 10.1111/dewb.12198 PMC6522326

[9] Kim H-W , Kankanhalli A. Investigating user resistance to information systems implementation: a status quo bias perspective. MIS Q 2009; 33 (3): 567–82.

[10] Myburgh H , Peters RP, Hurter T, Grobbelaar CJ, Hoddinott G. Transition to an in-facility electronic Tuberculosis register: lessons from a South African pilot project. South Afr J HIV Med 2020; 21 (1): 1025. 32158556 10.4102/sajhivmed.v21i1.1025 PMC7059247

[11] Khan SZ , Shahid Z, Hedstrom K, Andersson A. Hopes and fears in implementation of electronic health records in Bangladesh. *E J Inform Syst Develop Countries* 2012; 54 (1): 1–18.

[12] Draugalis JR , Coons SJ, Plaza C. Best practices for survey research reports: a synopsis for authors and reviewers. Am J Pharm Educ 2008; 72 (1): 11. 18322573 10.5688/aj720111 PMC2254236

